# Factors affecting residents transition from long term care facilities to the community: a scoping review

**DOI:** 10.1186/s12913-017-2571-y

**Published:** 2017-10-04

**Authors:** Shannon Freeman, Kristen Bishop, Lina Spirgiene, Erica Koopmans, Fernanda C. Bothelo, Trina Fyfe, Beibei Xiong, Stacey Patchett, Martha MacLeod

**Affiliations:** 1 School of Nursing, University of Northern British Columbia, 3333 University Way, Prince George, BC V2N 4Z9 Canada; 2 Faculty of Health Sciences, Health and Rehabilitation Sciences, Western University, 1151 Richmond St, London, ON N6A 3K7 Canada; 3 Department of Nursing and Care, Lithuanian University of Health Sciences, Mickevičiaus 9, -44307 Kaunas, LT Lithuania; 4 School of Health Sciences, University of Northern British Columbia, 3333 University Way, Prince George, BC V2N 4Z9 Canada; 5 School of Public Health, University of Sao Paulo, Dr. Arnaldo Street 715, Sao Paulo, SP 01246-904 Brazil; 6 Northern Medical Program, University of Northern British Columbia, 3333 University Way, Prince George, BC V2N 4Z9 Canada; 7 School of Nursing, Jilin University, 965 XinJiang Street, ChangChun, JiLin, 130012 China; 8 Department of Quality, Planning and Information, Northern Health, 543 Front Street, Quesnel, BC V2J 5K7 Canada

**Keywords:** Long-term care facilities, Home for the aged, Discharge planning, Older adults, Patient oriented care, Patient centred care

## Abstract

**Background:**

Long-term care facilities (LTCFs) are often places where persons with complex health needs that cannot be met in a community setting, reside and are cared for until death. However, not all persons experience continuous declines in health and functioning. For some residents who experience improvement in personal abilities and increased independence, transition from the LTCF to the community may be an option. This scoping review aimed to synthetize the existing evidence regarding the transition process from discharge planning to intervention and evaluation of outcomes for residents transitioning from LTCFs to the community.

**Methods:**

This review followed a five-stage scoping review framework to describe the current knowledge base related to transition from LTCFs to community based private dwellings as the location of the discharge (example: Person’s own home or shared private home with a family member, friend, or neighbour). Of the 4221 articles retrieved in the search of 6 databases, 36 articles met the criteria for inclusion in this review.

**Results:**

The majority of studies focussed on an older adult population (aged 65 years or greater), were conducted in the USA, and were limited to small geographic regions. There was a lack of consistency in terminology used to describe both the facilities as well as the transition process. Literature consisted of a broad array of study designs; sample sizes ranged from less than 10 to more than 500,000. Persons who were younger, married, female, received intense therapy, and who expressed a desire to transition to a community setting were more likely to transition out of a LTCF while those who exhibited cognitive impairment were less likely to transition out of a LTCF to the community.

**Conclusions:**

Findings highlight the heterogeneity and paucity of research examining transition of persons from LTCFs to the community. Overall, it remains unclear what best practices support the discharge planning and transition process and whether or not discharge from a LTCF to the community promotes the health, wellbeing, and quality of life of the persons. More research is needed in this area before we can start to confidently answer the research questions.

## Background

As populations continue to age across industrialized and emergent nations, the demand for more complex care is increasing. The growing number of frail older adults and vulnerable persons with disabilities necessitate a multidisciplinary response from across the care continuum. Symptom presentation is often ambiguous, threats to health multi-factorial, and trajectories of change highly variable with outcomes of care uncertain [1]. Consequently, the pressure on policy makers to respond to the care needs of vulnerable populations is growing [2, 3].

Older adults report better quality of life (QOL) when they are able to live and receive support in their preferred location of care [4]. Supporting persons to receive care supports matched to meet their own needs and provided in their chosen location may promote the person’s sense of control, empowerment, and improve QOL [5]. The majority of persons wish to remain in their own homes and have their needs supported in the community through a combination of formal and informal supports [6]. However, the resources and supports necessary to safely address increased levels of health complexity may not always be available in the community. For these persons, whose needs cannot always be met by available formal and informal supports, long-term care facilities (LTCFs) provide a necessary and appropriate care service.

Various terminologies are used to describe LTCFs including nursing homes, skilled nursing care facilities, personal care homes, residential care facilities, and long-term care homes. This terminology varies both within and across countries. For the purpose of this paper, the term LTCFs will be used and generally refers to care institutions that “serve diverse populations who need access to 24-hour nursing care, personal care and other therapeutic and support services” [7] that are not provided through home care programs, in retirement homes, in assisted living facilities, in the persons own home or in a shared private home. Generally, most LTCFs aim to provide a combination of medical, nursing, and social care in a residential care setting.

In Canada, the process to enter a LTCF differs across provincial and regional jurisdictions, but is primarily based on the complexity of clinical health needs of the person and level of dependency in addressing these needs. LTCFs support persons whose needs require more complex care than can be addressed in the community. Although persons who require more intensive levels of care exhibit complex needs and high dependency, they also may prefer to live as normal and unconstrained as possible [8, 9]. In this way, LTCFs are expected to provide resources to support the residents’ autonomy so they may be as active as they can and live their lives the way they want [10]. While the majority of persons exhibiting increased health complexity are older adults, advanced age (e.g. aged over 65 years) is not a requirement for eligibility to LTCFs in Canada.

While the majority of people wish to remain in their homes and be supported to live and die in the community [6, 11, 12], LTCFs in Canada are increasingly becoming places where older adults reside until death. Yet, not all older adults experience a continuous trajectory of decline in abilities following entry to LTCFs [13, 14]. Following entry to a LTCF some residents experience improvements in personal abilities and increased independence, becoming able to return home or transition to the community [15]. In this case, discharge planning involving multiple stakeholders is warranted [15]. In the United States (US), the duration of stay in a LTCF may be characterized as short and long-term stays [16]. Short-stay residents, who enter due to an acute episode often receive enhanced rehabilitation and care with an expected goal of transition to the community [16], account for more than 1 million discharges from LTCFs to community every year in the US [15, 17], a trend that has been reported over multiple decades. In contrast to the large number of discharges from LTCFs reported in the US, transition from LTCFs to the community in Canada is rare. A Canadian study of six provinces by Hirdes, Mitchell, Maxwell, and White found that less than 1% of LTCF residents transitioned to the community [18].

The definition of ‘transition’ varies according to disciplinary focus but the widespread definition involves how people respond to change over time [19]. Transition may be defined as “a process of convoluted passage during which people redefine their sense of self and redevelop self-agency in response to disruptive life events” [19]. Transition of care refers to the care a patient receives as they leave one care setting and are moved to another [20]. The American Geriatrics Society describes transitional care as “a set of actions designed to ensure the coordination and continuity of health care as patients transfer between different locations or different levels of care within the same location” [21].

Previous research has focussed upon transitions from hospital to other care settings including LTCFs [22, 23] and examined transitions from LTCFs to hospital or emergency departments [24]. However, to the knowledge of these authors, no scoping review has been conducted to critically examine the existing evidence about transitions from LTCFs to the community. Therefore, this article aimed to review the published scientific research studies about transition from LTCFs to the community to examine what is known about the discharge process and its associated factors by answering three main questions:

1. What are the characteristics of the residents who have been discharged from LTCFs to the community?
2. What are the associated factors surrounding resident discharge from LTCFs to the community?
3. What are outcomes experienced by residents post-discharge?

## Methods

The five-stage scoping review framework outlined by Arksey and O’Malley [25] was used to describe the current knowledge base related to transition from LTCFs to the community. These stages included: (1) identification of the research question, (2) identification of relevant studies, (3) study selection, (4) charting the data, and (5) collating, summarizing, and reporting the results. This scoping review methodology was selected for its capacity to “map relevant literature in the field” [25]. It also allowed researchers to draw conclusions on the overall state of research activity on a topic and suggest future directions for research [25]. Although scoping reviews do not address the quality of included sources, this method was appropriate for this emerging field of study and provided a rigorous framework to carry out a review systematically.

### Search strategy (identification of relevant studies)

Six databases were used to search for relevant literature on the transition from LTCFs to the community: SCOPUS, PubMed, CINAHL EBSCO, PsychINFO EBSCO, Embase OVIDSP and Web of Science ISI. The literature search was performed with the assistance of a research librarian at the University of Northern British Columbia in Canada. Articles published from January 1, 2000 to December 1, 2015 were selected to be both inclusive and relevant.

Search strategies were created for individual databases. These strategies involved various combinations of keywords and subject headings (when available). These terms included: *facility, residential home or care, nursing home, home for the aged, long term care, assisted living, convalescence,* and *home care, private dwelling or home, independent living, living alone,* and *discharge, transfer, exit, move or transition*. Searches were limited to English language articles only.

### Study selection

Inclusion criteria specified that included articles must 1) focus on resident transition from a LTCF; 2) include a community based private dwelling as the location of the discharge (e.g. Own home, shared private home with family member, friend, or neighbour); and 3) include persons over the age of 18. Articles exclusively studying persons discharged from acute care, hospital, or non-institutional setting were excluded from the review. Additionally, articles were excluded if the population in the study was discharged exclusively to communal or congregate setting (e.g. group home). Due to the changing nature of LTCFs both in terms of populations and regulatory policies and procedures, articles published prior to January 2000 were also excluded.

**Fig. 1:**
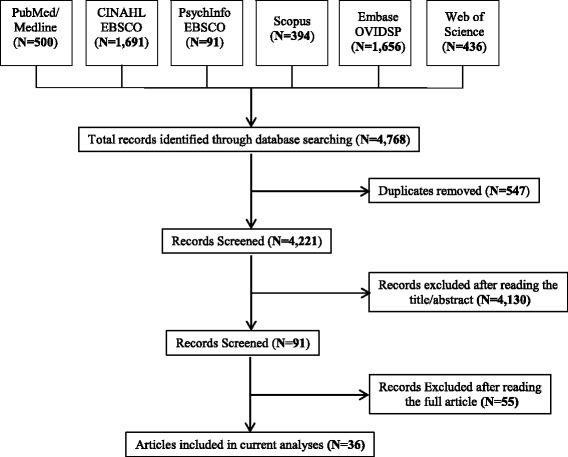
Scoping Review Flow Diagram of Article Selection Process

After screening titles and abstracts for relevancy, two authors reviewed full-text articles independently. If a discrepancy occurred amongst the reviewers, a third reviewer was consulted to make an inclusion or exclusion decision through collaboration and consultation. Interrater reliability noted 98.8% agreement between the two authors. A detailed article search chart outlining inclusion and exclusion data can be found in Fig. 1. From the original 4221 non-duplicate articles selected for review, 36 articles met criteria for inclusion in this scoping review. Microsoft Excel was used to categorize, extract and organize the data. Thematic and descriptive numerical analyses were conducted on all extracted data.

## Results

**Fig. 2:**
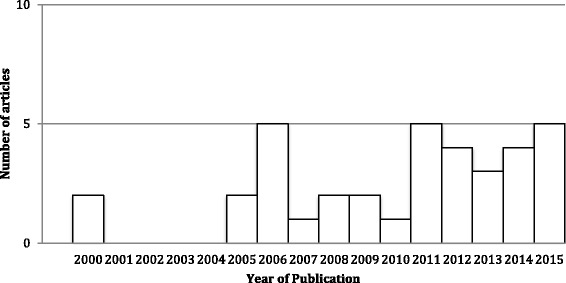
Articles Included by Year of Publication (2000–2015), *N* = 36

A summary of characteristic from the 36 articles can be found in Table 1. Study objectives and post-discharge outcomes are described in Table 2 while the purpose and main findings of all articles are presented in Table 3. With the exception of seven articles [26–32], the majority of articles focused on older adults (29/36), specifically those aged 65 or greater [33–47]. A few articles focused on specialized populations that shared a similar characteristic such as persons with an intellectual disability [30, 32, 48] or persons who had a stroke [26, 46]. One article focused on persons who had a common experience of a motor vehicle accident [33]. The majority of articles (30/36) included both males and females [26, 28, 30–37, 39–59]. The main focus for most articles (21/36) was on resident profiles [26, 28, 32, 33, 35, 36, 40–47, 51, 53–57, 60]. The study designs employed predominantly quantitative techniques [26, 28–30, 32–38, 40, 42–57, 60]. Research in this area represents a new and emerging field where over half of the included articles (22/36) were published in the last 5 years [26, 28, 29, 31, 35–38, 41–45, 47–50, 52, 54–56, 60] (Fig. 2).

**Table 1:** Description of studies characteristics background (*N* = 36)

Study Characteristic	Number of Studies	Study Characteristic	Number of Studies
Study Population:		Study Design:	
• Older adults	29	• Quantitative	30
• ≥ 65 years as inclusion criteria	15	▪ Longitudinal	17
• Average age ≥ 65 years	24	▪ Cross-sectional	5
• Person with intellectual disabilities	3	▪ Quasi-experimental controlled trial	1
• Persons with functional limitations	1	▪ Randomized control study	1
• Persons who had stroke	2	▪ Prospective cohort	3
• Persons with motor vehicle crash trauma	1	▪ Retrospective cohort	2
• Persons with specified conditions	1	▪ Case Control	1
Data Type:		• Mixed methods	4
• Primary	11	• Qualitative	2
• Secondary	19	Sample Size:	
• Both primary and secondary	6	• < 500	16
Study Focus:		• 500–10,000	12
• Persons only	21	• > 10,000	8
• Persons and one or more stakeholder(s)	13	Country of Study Origin	
• Institution and policy documents	4	• United States of America	30
Distribution By Sex:		• Australia	2
• Mixed sex-sample	30	• Finland	1
• Unspecified	6	• Germany	1
		• Lithuania	1
		• United States of America and Canada	1

**Table 2:** Objectives and outcomes of the discharge from a LTCF to a community setting (*N* = 16)

Author	Outcomes Measured Following Discharge from a LTCF	Main Findings Related to Post-Discharge from a LTCF
Aaland, Leffers & Hlaing, 2006	▪ Driving status ▪ Level of independence in feeding, expression, and locomotion	▪ Majority of older adults were able to regain functional activities after discharge from LTCF to community ▪ 52.2% continued to drive a motor vehicle post-discharge
Arling, Abrahamson, Cooke, Kane & Lewis, 2011	▪ Readmission during 1 year post-discharge	▪ 12% had 1 nursing home readmission and 2% had 2 or more in year post-discharge
Bardo, Applebaum, Kunkel, & Carpio, 2014	▪ Location status, State Medicaid waiver database and the nursing home MDS	▪ Most diversion and transition consumers who were still alive at the 6-month follow-up were living in the community
Callahan, Arling, Tu, Rosenman, Counsell, Stump, & Hendrie, 2012	▪ Transitions in care across all sites of care, outbound (transitioning out of the site), inbound (transitioning into the site), probability of a transfer between sites of care	▪ For transitions out the nursing facility, the conditional probability was higher to return home without formal services and to go to the hospital ▪ 30-day rehospitalization rate in older adults with dementia was 23%
Delate, Chester, Stubbings, & Barnes, 2008	Impact of medication reconciliation program on: ▪ Post-discharge mortality ▪ Rehospitalization ▪ Ambulatory clinic, emergency department visits	60 days after SNF discharge those in medication reconciliation group experienced: ▪ 78% reduction in risk of death ▪ Higher mean cumulative ambulatory care visits
Gassoumis, Fike, Rahman, Enguidanos, & Wilber, 2013	Discharge episodes and predictor variables: ▪ Sociodemographic ▪ Psychological ▪ Physical health ▪ Residential Facility ▪ Health care status	Those who transitioned to the community between 91 and 365 were more likely to have: ▪ Fallen within 180 days of admission ▪ Transitioned to and back from an acute care setting during the first 90 days of their episode.
Graessel, Schmidt, & Schupp, 2014	Living at home 2.5 years after discharge	75% of stroke survivors were still living at home 30 months after discharge ▪ Those with higher functional independence and health related quality of life at time of discharge continued to live at home more frequently than those with lower scores
Graham, Anderson, & Newcomer, 2005	Impact of program on transitions: ▪ Transitions in/across care sites ▪ Outcomes of program post-discharge	After 180 days: ▪ 30/36 who were discharged to the community remained at home ▪ 5/36 readmitted to a skilled nursing facility ▪ 3/36 died
Howell, Silberberg, Quinn, & Lucas, 2007	▪ Death ▪ Readmission to nursing home	▪ 72.6% of the entire sample of persons (*N* = 1354) remained in the community during the first year after leaving the nursing home ▪ 18.8% persons who were discharged died sometime during first year at home ▪ More men than women had a LTCF readmission
McCarthy, Szymanski, Karlin, & Katz, 2013	▪ Suicide rates 6 months post-discharge	▪ Suicide risk was 2.4 times higher overall and 2.3 times higher for males following discharge from VA nursing home compared to age and gender matched persons receiving care from the entire VA system ▪ Suicide risk greatest in first 3 weeks post-discharge then rest of 6-month post-discharge period
Mudrazija, Thomeer, & Angel, 2015	▪ Post-discharge living arrangements	▪ Women were more likely to live alone or with kin after discharge ▪ Men were more likely to live with a spouse or transfer to another institution
Robinson, Porter, Shugrue, Kleppinger, & Lambert, 2015	▪ Quality of Life ▪ Life Satisfaction ▪ Use of Health Services	▪ For the majority of respondents who remained in the community, quality of life and life satisfaction improved significantly after transition, and stayed high ▪ 14% returned to an institution one year after transition
Toles, Anderson, Massing, Naylor, Jackson, Peacock‐Hinton, & Colón‐Emeric, 2014	▪ Time to acute care utilization ▪ Emergency department visit ▪ Hospitalization	▪ 22.1% of older adults had an episode of acute care use within 30 days; 7.2% had an ED visit and 14.8% had a rehospitalization ▪ 37.5% had first acute care use within 90 days ▪ Health outcomes following discharge from a skilled nursing facility are multifactorial and relate to individual and system characteristics
Winkler, Farnworth, Sloan, & Brown, 2011	• Aimed to understand transition experience and identify key outcomes perceived by the participants	• 9 key outcomes identified: Improved independence (improved continence, getting around & movement, speaking, swallowing & eating), improved well-being (happier & less distressed, less difficult behaviour), and increased social inclusion (having things to do, being known in the community, family & friends)
Wysocki, Kane, Dowd, Golberstein, Lum, & Shippee, 2014	▪ First potentially preventable hospitalization experienced during observation period and first hospitalization of any type (preventable or non-preventable) ▪ Person’s group (stayer or nursing home transitioner)	▪ Transitioners had increased hazard of experiencing potentially preventable hospitalization by 40% over nursing home stayers ▪ Transitioners had 58% greater risk of experiencing any types of hospitalization than nursing home stayers
Young, 2006	▪ Adaptive and maladaptive behaviour ▪ Choice-making ▪ Objective life quality to reflect changes in skills and lifestyle after the institution.	▪ Both groups increased adaptive behaviours, choice-making and life quality in new residential location compared to in institution ▪ No change in level of maladaptive behaviour

**Table 3:** Purpose and main findings of all articles included in this scoping review (*N* = 36)

Study (Year)	Author institutional affiliations	Study Purpose	Main Study Findings/Study Conclusions
Aaland, Leffers, & Hlaing, 2006	Parkview Hospital, Indiana University-Purdue University	“The objective of this study was to evaluate if elderly patients discharged to a NH improved in their physical impairment related to trauma enough to be discharged from the NH or if the NH became a permanent address change” (p. 815).	▪ Hospital discharge to a NH following a motor vehicle accident trauma should be perceived as a transitional step back to a community setting ▪ At time of discharge from a NH, older adults were able to recover partial or full functional independence
Arling, Abrahamson, Cooke, Kane & Lewis, 2011	Indiana University, Western Kentucky University	“The aim of our study was to identify facility and market factors that influence the transition from nursing home to community to develop system-level interventions that complement our individuality focused return to community initiative” (p. 791)	▪ Multiple factors influence discharge from NH to the community including resident, facility, and market factors ▪ Residents entering Medicare oriented facility more likely to be discharged to the community and less likely to become long-stay NH clients
Arling, Kane, Cooke & Lewis, 2010	Indiana University, University of Minnesota, Minnesota Department of Human Services	“To analyze nursing home utilization patterns in order to identify potential targeting criteria for transitioning residents back to the community.” (p. 691)	▪ Majority of discharges (85%) occurred within first 30 days of admission ▪ Interventions for community transition should ideally occur between 90 and 120 days following first admissions ▪ Interventions should focus on residents who prefer discharge to a community setting
Arling, Williams, & Kopp, 2000	University of Missouri, Medicalodges	“Our objectives are to describe factors related to receipt of therapies at admission to the facility, and to examine three outcomes within 90 days after nursing home admission: community discharge, death, or remaining in the nursing facility”. (p. 588)	▪ Therapy use, when controlling for other covariates, was positively associated with community discharge and negatively with mortality ▪ Medicare was associated with access to therapy ▪ Therapy provision was influenced by both resident and staff view of potential for improvement in functional abilities
Bardo, Applebaum, Kunkel, & Carpio, 2014	Miami University, Arizona Health Care Cost Containment System, Scripps Gerontology Center	“to evaluate the effectiveness of Ohio’s Diversion and Transition Program, which was specifically designated for individuals who were 60 years old or older.” (p. 210)	▪ Barriers and promising practices were identified. ▪ Revealed innovative intervention strategies ▪ Most diversion and transition consumers still alive at 6 month follow up were living in the community
Brown, Raue, Mlodzianowski, Meyers, Greenberg, & Bruce, 2006	Stein Gerontological Institute, Miami Jewish Home and Hospital; Well Medical College of Cornell University	“To assess the completeness and accuracy of clinical information provided by referral sources to visiting nurses for patients admitted to receive home health care.” (p. 339)	▪ Essential clinical information often missing during transfer of older adult to home care sector ▪ 88.4% had medication discrepancies between in-home nurse review and admission information (*n* = 215) ▪ 34.9% lacked clinical information on medication allergies
Callahan, Arling, Tu, Rosenman, Counsell, Stump, & Hendrie, 2012	Multiple departments at Indiana University; Regenstrief Institute, Inc.	“To gain a more-complete understanding of the frequency and type of transitions in care of older adults with and without dementia, giving particular attention to transitions to and from nursing facility care over time.” (p. 814)	▪ Persons with dementia had greater Medicare and Medicaid nursing facility use, greater hospital and home health use, more transitions to care per person-year of follow-up, and more transitions.
Chen & Berkowitz, 2012	National Taiwan University; Columbia University	“The purpose of this study was to better understand the interplay between older adults’ home- and community based services and their residential transitions.” (p. 2)	▪ Use of different home and community based services and combinations thereof were associated with different directions in residential transitions
Delate, Chester, Stubbings, & Barnes, 2008	Departments of Pharmacy and Continuing Care, Kaiser Permanente Colorado; School of Pharmacy, University of Colorado at Denver and Health Sciences Center	“The purpose of this investigation was to assess the effectiveness of the KPCO medication reconciliation program after discharge from an SNF with regard to its impact on mortality, rehospitalizations, ambulatory clinic visits, and emergency department visits compared with usual care.” (p.445)	▪ Pharmacist-managed program resulted in 78% reduction in risk of death after SNF discharge ▪ Higher mean cumulative ambulatory care visits
Fries & James, 2012	University of Michigan	“Our intent was to enable states to improve Nursing Facility Transition (NFT) targeting strategies and thereby improve the use of scarce fiscal resources earmarked for transition activities.” (p.2) • To examine characteristics of long-stay residents discharged from nursing facilities • Identify target population of nursing home residents who may benefit from nursing facility discharge program • To create a discharge algorithm from the MDS 2.0	▪ Substantial differences observed by client length of stay for all characteristics tested ▪ Lower-acuity persons more prevalent among transitionees than among those remaining in the nursing facility
Gassoumis, Fike, Rahman, Enguidanos, & Wilber, 2013	Davis School of Gerontology, University of Southern California; Innovate50, San Francisco	“The purpose of this study is to: (a) examine natural patterns of discharge among NF residents in California, and (b) compare characteristics that predict community discharge among short-stay residents and long-stay residents.” (p. 77)	▪ Half of all admissions resulted in community discharge within 365 days (*n* = 1879; 49.9%) ▪ Over 90% of discharges to the community occurred within the first 90 days ▪ Transition to and back from acute care had greatest negative affect on potential to discharge while having a support person who is positive towards discharge had the strongest positive affect associated with discharge within the first 90 days ▪ Few characteristics predicted discharge to community for longer stay (>90 days) residents ▪ Cancer reduced odds of discharge 62% ▪ Severe cognitive impairment reduced odds of discharge 56% ▪ Resident preference to discharge had no effect after 90 days
Gozalo, Leland, Christian, Mor, & Teno, 2015	Department of Health Services, Brown University; Division of Occupational Science and Occupational Therapy, University of Southern California; Abt Associates Inc.; Providence Veteran’s Administration Medical Center	“To examine the effect of the relationship between volume (number of hip fracture admissions during the 12 months before participant’s fracture) and other facility characteristics on outcomes.” (p.2043)	▪ Overall rate of successful discharge to the community was 31% within 30 days of discharge from the hospital ▪ Participants discharged to high-volume SNFs (all else equal) were approximately twice as likely to achieve successful discharge to the community
Graessel, Schmidt, & Schupp, 2014	Department of Psychiatry and Psychotherapy, Center for Health Services Research in Medicine, Friedrich-Alexander-Universitaet Erlangen-Nuernberg; Erlangen-Nuernberg Department of Neurology and Neuropsychology, Clinic for Specialized In- and Outpatient Rehabilitation Medicine, Herzogenaurach, Germany	“To determine whether stroke patients’ functional status or health-related quality of life would predict whether they lived at home 2.5 years after discharge from neurological inpatient rehabilitation.” (p.212)	▪ 30 months after discharge, 75% of the stroke survivors were still living at home. ▪ Patients continued to live at home significantly more frequently when they had fewer mortality-relevant comorbidities, higher BMI and higher increase in functional independence
Graham, Anderson, & Newcomer, 2005	University of California, Berkeley; Contra Costa County Aging and Adult Services; University of California	“This article describes [Providing Assistance to Caregivers in Transition] PACT’s features and the issues affecting its success during its initial 24 months of operations. Among these are recruitment, enrollment, and participant and staff perceptions about the value of the program.” (p.93)	▪ During first 2 years, 38/42 opened cases were assisted to discharge to the community ▪ Feedback of the program by caregivers indicated satisfaction with instrumental and emotional support provided through the PACT program ▪ Most PACT participants (*n* = 38) remained in the community after 180 days
Holup, Gassoumis, Wilber, & Hyer, 2015	Florida Policy Exchange Center on Aging, School of Aging Studies, University of South Florida; Davis School of Gerontology, University of Southern California,	“Examines the influence of facility characteristics on the transition of nursing home residents to the community after a short stay (within 90 days of admission) or long stay (365 days of admission) across states with different long-term services and supports systems.” (p.1)	▪ Facility characteristics, including size, occupancy, ownership, average length of stay, proportion of Medicare and Medicaid residents, and the proportion of residents admitted from acute care facilities are associated with discharge ▪ Facility characteristics were more strongly related to community discharge than the characteristics of the markets in which they were located
Howell, Silberberg, Quinn, & Lucas, 2007	Rutgers Center for State Health Policy; Duke University Medical Center; New Jersey Department of the Public Advocate, Division of Elder Advocacy, Trenton, NJ; Institute for Health, Health Care Policy, and Aging Research, Rutgers University	“To inform states with nursing home transition programs, we determine what risk factors are associated with participants’ long-term readmission to nursing homes within 1 year after discharge.” (p. 535)	▪ At time of one year post-discharge: 72% continued to live in community, 18.8% had died, and 8.6% readmitted to a nursing home for >90 days ▪ Multiple factors significantly associated one year post-discharge outcomes included: sex, health beliefs, living situation, informal/formal assistance with ADLs, transportation, medication managements, ADL/IADL abilities, and post-discharge acute health events (e.g., falls, ED visits or hospitalizations)
Marcum & Hardy, 2015	Division of Geriatric Medicine, School of Medicine, University of Pittsburgh; Summit ElderCare, Worcester, Massachusetts	“Objective of this pilot study was to describe potential medication management deficiencies of older SNF residents transitioning home.” (p.1267)	▪ Medication management deficiencies were found to be common in a high-risk group of elderly adults making this important transition
Martikainen, Moustgaard, Murphy, Elinio, Koskinen, Martelin, & Noro, 2009	Department of Sociology, University of Helsinki, Finland; London School of Economics, UK; National Institute for Health and Welfare, Helsinki, Finland	“The focus of this article is on the effects of three important sociodemographic factors – living arrangements, housing tenure, and household income – on entry to and exit from long-term institutional care … Our specific aims were to (a) assess how gender, age, living arrangements, housing tenure, and household income are associated with the risk for entry into and exit from long-term institutional care, through either death or return to the community; (b) assess whether these associations are independent of each other and mediated by health status; (c) evaluate, in particular, why gender is associated with institutionalization and how it modifies the impact of other variables; and (d) estimate the mean number of days spent in institutional care.” (p. 36)	▪ At time of 5 year follow-up, 28.4% of men and 28.1% of women had been discharged to the community ▪ Factors that increase risk to enter long-term care are same risk factors for exit from long-term care but associations were weaker and in the opposite direction (with exception of age).
McCarthy, Szymanski, Karlin, & Katz, 2013	Department of Veterans Affairs; Yale School of Public Health	“Evaluated suicide rates following discharge from Veterans Affairs nursing homes … Evaluated measures of serious mental illness, depression, dementia, behavior problems, and pain as predictors of suicide after discharge.” (p. 2261)	▪ Suicide risk was elevated following 6 months post-nursing home discharge ▪ Suicide risk was 2.4 times as high overall and 2.3 times as high for men (p. 2264)
Meador, Chen, Schultz, Norton, Henderson, & Pillemer, 2011	Cornell University; Community Health Foundation of Central and Western New York;	“This article describes barriers to nursing home discharge encountered in an intervention designed to transition nursing home residents to the community.” (p.2)	▪ No differences found between social, demographic, and health characteristics of the person ▪ Barriers to discharge from nursing home to the community included a)level of medical complexity; b)family and social support, and c)availability of appropriate housing in the community((page 10) ▪ Staff knowledge of transition process and network of contacts helped simplify and streamline discharge process for residents
Mudrazija, Thomeer, & Angel, 2015	Edward R. Roybal Institute on Aging, School of Social Work, University of Southern California; Department of Sociology, University of Alabama at Birmingham; Lyndon B. Johnson School of Public Affairs, The University of Texas at Austin	“To identify the ways in which gender influences likelihood of discharge from LTC facilities, duration of stay in LTC facilities, and post-discharge living arrangements.” (p.442)	▪ Women are more likely than men to be discharged from LTC facilities during the first year of stay. ▪ Women are more likely to live alone or with kin after discharge, whereas men are more likely to live with a spouse or transfer to another institution. ▪ Gender differences in the availability and use of family support may partly account for the gender disparity of LTC discharge and post-discharge living arrangements.
Naomi, Shiroiwa, Fukuda, & Murashima, 2012	The Japan Baptist Hospital; Ritsumeikan University; University of Tokyo	“To examine the effectiveness of deinstitutionalizing the disabled elderly with the aim of cost reduction.” (P. 1) “This study examines the effectiveness of the deinstitutionalization of disabled elderly individuals for the purpose of cost reduction, and clarifies the policy issues surrounding an aging society.” (p.3)	▪ 87/139 patients considered candidates for deinstitutionalization at discretion of care managers and home visiting nurses ▪ Estimated home care costs were higher than institutional care costs ▪ Deinstitutionalization of the elderly did not reduce healthcare costs. (p. 9)
Newcomer, Kang, & Graham, 2006	University of California, San Francisco; University of California, Berkley	Evaluate the effectiveness of the Providing Assistance to Caregivers in Transition (PACT) program. “We compare nursing home discharge rates and length of stay between those individuals in the intervention group and those in the usual care or control group” (p. 385–386)	▪ No statistical differences observed in the discharge rate (84% treatment vs 76% controls) or in the median length of stay (42 days vs 55 days) between the intervention and control groups
Nishita, Wilber, Matsumoto, & Schnelle, 2008	University of Southern California; Vanderbilt Center for Quality Aging, Nashville, Tennessee.	“To examine nursing facility residents’ or their legal proxies’ perspectives on transitioning out of nursing facilities by assessing residents’ perceptions of their ability to live more independently, their preferences regarding leaving the facility, and the feasibility of transitioning with community support.” (p.1)	▪ 23% believed resident had ability to transition ▪ 46% indicated preference to transition ▪ After discussions of potential living arrangements and supports, 33% thought transition was feasible ▪ More residents who preferred transition were identified through screen than through MDS 2.0
Penrod, Kane, & Kane, 2000	University of Nebraska; University of Minnesota	“This study examines the effect of family caregiving on the probability that nursing home residents would be discharged to the community within 6 weeks following nursing home admission” (p.66)	▪ 34% of residents went home by 6 weeks post-hospital discharge ▪ Informal care provision (not solely presence of a caregiver visiting daily) may increase the quality and amount of care residents receive thus influencing rehabilitation outcomes and return to home ▪ Caregiver advocacy may signal a well-functioning support system
Poole, Duvall, & Wofford, 2006	College of Social Work, University of South Carolina,	“Our study is the first application to planning and evaluation of a state nursing home-to-community transition project. We used three research questions to guide this component of the evaluation during implementation of the CARS project: 1) What key elements or conceptual domains must be addressed to help a person with physical disabilities move out of a nursing facility successfully? 2) How important is each element or domain? 3) To what extent does the Texas CARS project address these elements or domains?” (p.12)	▪ Resulted in a visual statistical model of 14 key conceptual elements that they deemed essential in a nursing home-to-community transition project ▪ Community participants reported that strategic components of the state project generally fit well with their perception of the ideal transition model ▪ Original study did not advance knowledge very far beyond observations and findings reported in the literature
Robinson, Porter, Shugrue, Kleppinger, & Lambert, 2015	Center on Aging at University of Connecticut Health Center; Division of Health Services, Connecticut Department of Social Services	“Investigated long-term outcomes of transitioning from institutions to community living over six years of the Money Follows the Person program, 2008–14, in Connecticut.” (p. 1634)	▪ For majority of participants, quality of life and life satisfaction improved significantly after transition ▪ About half of the participants visited hospitals or emergency departments after transition ▪ Only 14% returned to an institution one year after transition ▪ Predictors of re-institutionalization included some not previously observed: mental health disability, difficulties with family members before transition, and not exercising choice and control in daily life.
Seekins, Ravesloot, Katz, Liston, Oxford, Altom White, Petty, & Kafka, 2011	University of Montana Rural Institute; Topeka Independent Living Resource Center; Association of Programs for Rural Independent Living; University of Kansas; ILRU at TIRR Memorial Hermann	“The objectives of this research were to: (1) assess the levels of nursing home emancipation services and barriers to nursing home transitions, including the role of secondary health conditions, and (2) to assess nursing home transition policies and procedures.” (p.245)	▪ Transitioned 2389 residents from nursing homes back to community living arrangements ▪ Only 4% returned to a nursing home for any reason
Spirgiene, Routasalo, & Macijauskiene, 2013	Lithuanian University of Health Sciences; University of Helsinki	“To examine residents’ resources for potential transition to the community after entry to LTCFs.” (p.523)	▪ 1/3 of residents preferred transition back to the community ▪ Many residents had resources (e.g. 10% had no cognitive impairment; 40% were ADL independent; 2/3 would feel safe in the community), yet none were involved in a discharge process due to lack of established nursing/social care services and transitional care plans ▪ A population of residents with no cognitive or functional impairments resided in long-term care facilities who were suitable candidates for transition back to the community
Thomas, Gassoumis, & Wilber, 2010	University of Southern California	“To determine the effect of a Social Health Maintenance Organization (S/HMO) on diverting older adults admitted into a nursing facility from converting to long-stay placement” (p.333)	▪ After controlling for selected sociodemographics, comorbidities, behavioral issues, mental health conditions, and other risk factors, being enrolled in the S/HMO increased the likelihood of successful discharge by 26%
Thorn, Pittman, Myers, & Slaughter, 2009	Pinecrest Supports and Services Center, United States; University of South Florida,	“Our aim was to design a functional system in a large residential facility that would increase community integrated learning opportunities. This was conducted with particular focus on increasing successful transitions to community-based living settings.” (p.893)	▪ “Highlights the advantages of creating a therapeutic milieu fostering learning and practicing functional skills in real-life activities and how this translates to increased community integration success for individuals with significant ID.” (p.899) ▪ Significant increases in the areas of community presence, community participation, community integration and community inclusion through community integrated learning opportunities.
Toles, Anderson, Massing, Naylor, Jackson, Peacock‐Hinton, & Colón‐Emeric, 2014	University of Northern Carolina; Duke University; University of Pennsylvania; Veterans Affairs Medical Centre	“To describe the time to first acute care use (e.g. emergency department (ED) use without hospitalization or rehospitalization) for older adults discharged to home after receiving post acute care in skilled nursing facilities (SNFs);to identify predictors of first acute care use.” (p.79)	▪ Post SNF discharge to the community, 22.1% of older adults used acute care within 30 days; 37.5% within 90 days ▪ Medicare beneficiaries had high use of acute care services post-SNF discharge ▪ Factors associated with acute care use are potentially modifiable
Winkler, Farnworth, Sloan, & Brown, 2011	Summer Foundation (Australia); Monash University, Australia; OT/Neuropsychologists from Osborn, Sloan, & Associates (Australia)	“To explore the transition experiences of young people with acquired brain injury who have lived in age care facilities and moved to the community-as well as the perspectives of their significant carer/carers. The study aimed to understand the outcomes of transition from residential aged care to the community; to identify key outcomes from their perspectives.” (p.155)	▪ A range of positive outcomes were identified resulting from transition from aged care settings to the community including increased independence, improved wellbeing and greater degree of social inclusion ▪ Environmental factors were critical to facilitating a positive outcome
Wodchis, Teare, Naglie, Bronskill, Gill, Hillmer, Anderson, Rochon, & Fries, 2005	Toronto Rehabilitation Institute; Institute for Clinical Evaluative Sciences; University of Toronto; University Health Network; Baycrest Centre for Geriatric Care; University of Michigan; Veterans Affairs, Ann Arbor, MI	“To determine the relation between rehabilitation therapy (RT) intensity and time to discharge home for stroke patients in skilled nursing facilities (SNFs).” (p.442)	▪ Rehabilitation therapy increased the likelihood of discharge to the community for all groups except those expected to be discharged within 30 days.
Wysocki, Kane, Dowd, Golberstein, Lum, & Shippee, 2014	Center for Gerontology and Healthcare Research, Brown University; School of Public Health, University of Minnesota; Department of Social Work and Social Administration and Sau Po Center on Ageing, The University of Hong Kong,	“To compare hospitalizations of dually eligible older adults who had an extended Medicaid nursing home (NH) stay and transitioned out to receive Medicaid home- and community-based services (HCBS) with hospitalizations of those who remained in the NH.” (p.71)	▪ Persons who transitioned from the nursing home to Medicaid home- and community-based services had a greater risk of hospitalization.
Young, 2006	School of Medicine, The University of Queensland, Mayne Medical School (Australia)	“To monitor changes in skills and life circumstances as residents of an institution that was to be permanently closed were progressively relocated into either dispersed homes in the community or cluster centres and to record any changes in adaptive and maladaptive behaviour, choice-making and objective life quality.” (p.421)	▪ Community group had a greater number of significant improvements and achieved more domestic skills in cleaning, laundry, table setting, food preparation and other routine household chores. ▪ Community group had significantly improved levels of trustworthiness and decreased sexual behavior ▪ Community group had significantly increased opportunities for everyday choice-making ▪ Both groups experienced improved objective quality of life in all areas measured over 2 years of living in the new location.

Study sample sizes had a large range from less than 20 participants [31] to more than 500,000 participants in the largest sample [37]. Almost all studies were conducted in the US [27–30, 33–39, 41, 42, 44, 45, 47–55, 57–61]. Non-US research in this area is scarce [26, 31, 32, 40, 43, 46, 56] and only one article used data gathered from more than one country [32, 46]. Authors’ background and type of institution varied across studies. Single academic institutional authorship was most prevalent (21/36) [26–28, 30–34, 36, 41, 44, 48, 51–55, 57, 58, 60, 61], of which five also included author partners from community locations such as hospitals and LTCFs [33, 34, 51, 56, 60] and five included research institutes, centres for aging, and non-profit partners [28, 30, 31, 36, 52]. Just over one-third of articles (14/36) were inter-institutional collaboration [29, 35, 37, 38, 40, 42, 43, 45–47, 50, 51, 56, 59] of which six included additional community and research institutional partners [29, 37, 46, 49, 50, 56] and four were multi-national collaborations [35, 40, 43, 46].

### Terminology

Terminology used varied across studies. When describing the process of leaving the LTCF and returning to the community, terminology to describe the transition included: discharge [26, 27, 31–33, 37, 39, 41–43, 45–47, 53–57, 59–61], community discharge [36, 38, 40, 44, 49–51], community transition [36, 38, 42, 49, 50], transition [27, 28, 30, 31, 34, 35, 41–43, 47, 48, 52, 60], nursing facility transition [54], nursing home transition [29, 39, 49, 57], return to the community [28, 37, 38, 40], return to community [41], deinstitutionalizing [56], transitioning [27, 28, 58], emancipation [29] and relocation [32].

Study locations also varied. LTCFs were referred to as long-term care facilities [43], nursing homes [26–29, 33–36, 38, 39, 41, 42, 45–47, 49–52, 54–59, 61], nursing facility [27, 36, 41, 44, 48–52, 54, 58], skilled nursing facilities [37, 45, 46, 53, 60, 61], long-term institutional care [40, 50], long-term care welfare facilities [56], long-term care medical facility [56], long stay placement [44], residential facility [30], aged care facility [31] and long-term institutions [32]. Only a few studies provided information about the size of the institution, such as the number of beds, average occupancy rates or physical dimension of the facility [30, 38, 45, 48, 49, 61].

### Theoretical framework or approach

Explicit mention of the theoretical perspective use to guide the research in this field was rare; only three articles mentioned a theoretical framework in their study or analysis. The theoretical frameworks mentioned included: an ecological model of health behaviour to investigate characteristics of facilities that influence transition to the community [38]; and Anderson’s model for health care [62, 63] to select and classify variables that might influence the risk of LTCF readmissions [39, 42]. Two others specified conceptual approaches in their studies: conceptual mapping technology developed by Trochim [64] was used to conceptualize key elements or theoretical domains of an intervention plan [27]; and Grounded Theory [65] to analyse qualitative data [31].

### Planning for discharge from LTCFs

Approximately one-third of the articles (14/36) described a discharge initiative. Some studies used programs [28, 39, 52–54, 57, 61], such as The Ohio Diversion and Transition Program [52] or New Jersey’s Nursing Home Transition Program [39]. Some articles described a discharge initiative organized as a project (i.e. New York State Department of Health’s “Project Home” [41]), a plan (i.e. post discharge home care plans [56]) or study interventions (i.e. cluster centre and community living compared to residential institutions [32]) [27, 29, 30, 32, 41, 44, 56]. Discharge initiatives were limited to local and regional action with the exception of one, which targeted a collection of nursing facility transition programs across multiple US states [54].

The length of stay residents experienced prior to their transition out of LTCF’s differed across studies. Some articles focussed on transition programs for residents who had resided in the facility for a short period of time (approximately 90 days) [44–46, 48, 49, 51, 57, 61]. Other studies focussed exclusively on residents with longer stays (generally greater than 100 days) [30, 32, 40, 43, 47, 54, 56, 58]. Several studies mentioned very long stays [30, 32, 40, 43, 56], with average lengths of stay greater than 2 years. Some articles included both short and long stays in LTCFs [33, 36, 38, 39, 50, 55].

### Transition to community location

Studies reported a variety of locations to which residents transitioned. Some focused directly on transition to community [27–31, 34, 35, 37–43, 46, 47, 49, 50, 53–58, 60, 61]. Of these, six articles specified that the transition to the community was accompanied by community-based support services [27, 30, 31, 35, 47, 49] and only three articles provided more information specifying the type of community location [35, 42, 56]. Interestingly, some studies examined residents as they transitioned across the care continuum starting from the hospital and traced patient transitions to LTCFs and then followed the same patient’s as they transitioned to the community [33, 45, 59]. In contrast, other studies included discharge from LTCFs to multiple locations of care, including the community (private residence with home health, private residence without home health) as well as other locations including group homes, assisted living, other nursing home and an acute-care hospital [36, 44, 50, 52]. Finally, one article did not focus on the discharge, but on all transitions among different locations of care [48]. This study included the transitions of residents among places including home/community, home without formal services, home with formal services, nursing facility, skilled nursing facility and hospital.

Five studies emphasized the importance of person-centred care to tailor the transition process to meet the individual needs of the resident [27–29, 41, 43]. Findings from the “Project Home” intervention emphasized that a person-centered care approach that “includes responsiveness to individual needs, the flexibility to match those needs with creative solutions, and the coordination of service providers, is a feasible and effective way to maximize positive outcomes for older people who want to live at home.” [41]. Furthermore, person-centered care planning activities may not only inform tailoring of care plans but may also serve as a foundation for effective care team collaboration during the transition between the LTCF and community care providers [43].

### Objectives and outcomes

Details describing study objectives and post-discharge outcomes are described in Table 2. Less than half of the articles presented post-discharge outcomes [26, 28, 31–33, 36, 39, 45, 47–49, 52, 53, 55, 61], of which few were described in depth. Transition outcomes examined included length of time residents were able to remain in the community post-transition, readmission to a nursing facility, hospitalization and mortality.

### Characteristics of residents who had transitioned out of LTCFs

Considering the individual determinants of health services utilization [63], characteristics of the persons residing in LTCFs potentially eligible for discharge should be considered key to the transition process. The needs identified by articles included in this review were primarily individual determinants. Residents residing in LTCFs were most likely to transition out of LTCFs if they were younger [58], married [36, 51], female [36, 42], had Medicare coverage [51], experienced daily pain [51], participated in or received intense therapy [51], were responsible for their own decisions [36] and preferred return to the community [36, 39, 50]. Residents who had recently had fractures were also more likely to return to the community [44, 45]. In contrast, residents were less likely to be discharged to the community if they had cancer [36] or were cognitively impaired [36].

Other facilitators important to transition included: independence of the persons in cognitive and functional abilities [33, 39, 43, 50, 56, 58], medical stability [39] and fitting a community discharge profile [50]. Furthermore, persons were more likely to transition out of a LTCT if they received independent living training or community skills training [27, 36], caregiver support [27, 36, 50], medication management [60] and community supports were made available to assist the person in activities of daily living [43, 49].

### Stakeholders involved in the transition

Discussion of the roles various stakeholders played during the transition decision making and care planning process was lacking. Less than one third of articles identified which formal care providers were involved. Of the articles that described the involved stakeholders, a wide variety of formal care providers were mentioned including: case managers, discharge planners, counsellors, residents, proxies, families, staff members and researchers. Interestingly, only one study described involvement of a care team involving stakeholders across the care continuum representing the LTCF, the community, and the person to be transitioned [57], whereas the other articles described involvement of stakeholders from only one or two locations of care.

Half of the articles (20/36) clearly mentioned informal caregivers [26, 27, 31, 33, 39–43, 45, 47–49, 51, 52, 56–59, 61], yet only two of them defined the role of the caregiver as “providing the most assistance with care or arranging care in the 6 weeks after the hospital discharge” [59] and “help family members or friends get what they need to live at home or in another community-based setting” and “participate in discharge planning” [27].

### Factors associated with the transition journey

A wide range of key risk and protective factors were identified at different times, from the time of “pre-discharge planning” (considering characteristics which would enable or deter the identification of potential for discharge to the community and/or influence the process to develop the discharge plan), through the “discharge process” (the transition of leaving the LTCF and returning to the community), and finally examining outcomes “post-discharge” (factors affecting the degree of discharge success and sustainability). The factors identified across these three phases in the discharge journey are described by individual, institutional, and community levels of needs and characteristics below.

#### Factors associated with ‘pre-discharge planning’

In the ‘pre-discharge planning’ phase, independent living training or community skills training [27, 36], caregiver support [27] and medication management [60] emerged as important individual level needs. At the institutional level, case management support, transitional assistance, administrative procedures, assessment, discharge planning processes and nursing facility collaboration were identified as necessary characteristics to support a plan for a discharge process. Facility characteristics including size, occupancy, ownership, average length of stay, proportion of Medicare and Medicaid residents, and the proportion of residents admitted from acute care facilities [38] were all factors, which influenced the likelihood of resident discharge to community. At the community level, education, advocacy, housing, consumer centred planning policy and funding opportunities [27] were identified as critical to inform whether discharge was feasible.

#### Factors associated with the transition process

To support the transition process, individual need characteristics identified as facilitators of discharge included independence of the persons in cognitive and functional abilities [33, 39, 43, 50, 56, 58], the person’s preference for discharge to the community [39, 50], medical stability [39] and fitting a community discharge profile [50]. Only one article [31] identified institutional level characteristics for transition process which included support provided by LTCF staff, consistency of LTCF care, and staff respect and dignity for the person. Community characteristics aiding the transition process included support for community discharge by a family member or other person close to the person [36, 50] and availability of community supports to assist persons in management of activities of daily living [43, 49].

Factors affecting the degree of transition success or outcomes post-discharge are limited. Receiving care in a LTCF with more licensed practical nurse hours per resident day [45], and longer duration of skilled nursing facility treatment [45] resulted in lower risk of acute care use post-discharge. Other institutional characteristics, which positively influenced post-discharge outcomes, included residing in LTCF with high percentage of admissions receiving therapy [51] and higher rehabilitation therapy intensity [46], as well as LTCFs with high investment in nursing staff [49] and high volume skilled nursing facilities [37].

### Recommendations for further study

Most articles highlighted existing inconsistences in the knowledge base and areas where further research is warranted. Recommendations for better understanding of the complexity of the discharge process, included further evidence summarizing factors and barriers that can influence the discharge such as: environment circumstances, proxy relationships, personal preferences, family and informal caregivers, nursing home staff and other health-care providers [27, 28, 35, 37, 38, 45, 48, 51, 52, 58, 59]. Moreover, further research was suggested to expand understanding of outcomes following the discharge [30, 31, 33, 42, 48, 51, 55]; and evaluation or improvement of existing discharging programs or initiatives [34, 42, 44, 50, 51]. Recommendations specific to study methods and protocols included more rigorous study methodology (increased sample size, replication across geographic regions) [27, 36, 45, 51, 53] and the requirement for expansion of study results [32, 39, 45, 60]. There was also a need to better understand the influence of quality and intensity of health care provided during the pre-discharge planning process [46, 51, 56].

## Discussion

Study findings highlight the heterogeneity and paucity of research examining transition of persons from LTCFs to the community, especially when considering the international context. Overall, the generalizability of findings in this scoping review remains greatly hindered as most were conducted in small geographic areas across the US and were led by single academic institutions. The majority of studies focussed on older adult populations with a patchwork of evidence on special populations with a shared health condition/diagnosis (i.e. stroke or acquired brain injury) or shared client experience (i.e. traumatic life event). Overall, it remains unclear what mix of multidisciplinary team members and institutional factors best support discharge planning and transition process. And finally, due to a lack of studies measuring post-discharge outcomes, it remains unclear from the current body of evidence whether discharge from a LTCF to the community promotes the health, wellbeing and quality of life of those who transition out of LTCFs and whether these transitions are cost-effective for the health care system. More research is needed in this area before we can start to confidently answer the research questions.

No gold standard emerged for the best time to identify and engage persons who may be eligible for transition out of a LTCF to the community in discharge planning activities and processes. Variation in the association between length of stay in a LTCF and potential for discharged was observed. For example, Arling [50] indicated that 85% of discharges occurred within the first 30 days of admission while Gassoumis [36] noted that the percentage of persons discharged increased to 90% within the first 90 days of entry into LTCF [36]. Interestingly, Gassoumis [36] also noted that a resident’s preference to discharge showed no effect on discharge to community after a 90 day or greater length of stay.

Considering the individual determinants of health services utilization [63], characteristics of the persons residing in LTCFs potentially eligible for discharge should be considered key to the discharge process. The needs identified in this review were primarily individual determinants. Residents residing in LTCFs were most likely to transition out of facilities if they were younger [58], married [36, 51], female [36, 42], had Medicare coverage [51], experienced daily pain [51], participated in or received intense therapy [51], were responsible for their own decisions [36] and preferred return to the community [36, 39, 50]. While demographic characteristics such as gender and age may be associated with the discharge process they were not recognized consistently as the strongest factors predicting discharge to the community. Other important facilitators of discharge included independence of the persons in cognitive and functional abilities [33, 39, 43, 50, 56, 58], medical stability [39], and fitting a community discharge profile [50]. Furthermore, persons were more likely to transition out of a LTCT if they received independent living training or community skills training [27, 36], caregiver support [27, 36, 50], medication management [60] and community supports were made available to assist persons in management of activities of daily living [43, 49].

In contrast to Poole who noted that “the need for person or ‘consumer-centered’ planning is widely cited in the transition literature” [27], findings in the current study suggest it may be less commonly discussed. Less than 15 % of articles (5/36 articles) in the current study discussed person-centered care. Although all of the number of studies were few, those that did discuss patient centered care emphasized the crucial role that person centered care planning plays to ensure the needs and preferences of the resident are heard and respected, Case managers and discharge planners play an important role to support patient centred care, provide education to the person and caregivers, and promote communication among multidisciplinary teams during transitions across the care continuum [66, 67]. Findings from the Project Home intervention noted that when a flexible organizational structure exists that empowers its case managers to advocate for client goals and preferences, then a person centered approach to care may be realized [41]. To ensure the person transitioning out of the LTCF is able to improve their health and QOL, it is important that the goals, values, and preferences of the resident be considered and incorporated throughout the discharge process. When the person residing in a LTCF, who is identified as a candidate for transition back to the community, is not the centre of the person-centred plan for care, the discharge care plan may be unsuccessful [68]. Considering this, further research is needed to examine best practices in patient centred care planning practice during the transition (discharge) process from planning to intervention and evaluation for residents transitioning from LTCFs to the community.

This scoping review identified a lack of focus on positive post-discharge outcomes such as measuring the impact of discharge on the resident and their informal caregivers’ health and QOL. Only one study [28] measured QOL and life satisfaction as post-discharge outcomes. Robinson et al. [28] found that QOL and life satisfaction improved for the majority of respondents who transitioned from an institution to the community. An interesting post-discharge outcome related to QOL was suicide rates post-discharge. One study revealed that suicide risk was significantly elevated following discharge from a Veteran Affairs (VA) nursing home compared to matched persons receiving care from the VA system [55]. This study indicated that suicide risk was greatest in the first 3 weeks post discharge [55]. While this study was limited to a population of older veterans it is an important finding as suicide can be indicative of life dissatisfaction [69] and warrants further research.

Other studies focussed on measureable available outcomes such as death, rehospitalization, or readmission without describing in depth the role that the discharge process had in relation to the outcome. Meaning, if the person died, the research did not describe in detail how the transition related to that death. It would be beneficial for future research to investigate whether outcomes which occurred post-discharge would not have occurred or have been preventable had the person not transitioned out of the LTCF. LTCF readmissions and their predictors, important measures at post-discharge, are used to plan interventions and allocate resources [39]; however it is also essential to look deeply at both positive and negative benefits that transition to the community may evoke. Gains obtained with transition to the community may be related to independence, well-being and social inclusion [31]. However, more research examining what occurs after discharge is a topic where more studies are needed [33, 48].

To determine the degree to which the post-discharge goal to improve the person’s health, wellbeing, and QOL was attained, outcome measurements should include both objective and subjective measures. While the majority of studies used individual level data and described a focus on the person, there was a lack of reporting of person-centred goals, values and preferences in relation to discharge and post-discharge outcomes. Instead, articles in the current review focused on measurable outcomes such as death, rehospitalization or readmission without describing in depth the person’s perspectives on improvements in their health, life satisfaction or QOL. It remains unclear from the current evidence available whether person-centred goals can be obtained post-discharge. Further research in this area should focus on measuring whether persons were able to receive post-discharge care supports in their new location, which were matched to their person-specific need. Moreover further research should examine whether the transition was successful to promote the person’s sense of control, empowerment, and improve their QOL as had been noted by Fry [5].

Finally, the needs to transition related to discharge planning for residents should include the needs and preference of informal and formal care supports that will be caring for persons in the community. Of the articles included in this review, fewer than half of the articles described the perspectives of stakeholder’s involvement in the transition. There remains a gap in understanding regarding the availability of and eligibility for community based care services and supports. These articles highlight a gap in our understanding of post-discharge outcomes and whether or not current transition programs are successful from the perspective of the person of focus and their circle of care.

Due to the emerging nature of research in this field, a systematic review is not yet warranted. Among the reviewed articles there was a lack of generalizability due to variation regarding the location as most articles were limited local studies and were conducted in the US. The US health system organization is unique, well-marked by Medicare and Medicaid programs for older adults and low-income people [70], making the findings obtained very specific and concentrated, and their use difficult for the international scientific community. It is unclear whether study findings would be applicable in other health care systems such as the Canadian publically funded system [71], Switzerland’s universally mandated private insurance system [71] or a two-tier system such as Japan’s [71]. Lack of generalizability of research findings necessitate further research and replication of studies [45] in this area to better understand processes and outcomes regarding discharge from LTCFs.

Articles reviewed in this study presented many recommendations for future research acknowledging inconsistences in the knowledge base and highlight areas where more research is needed. Gaps identified in currently available research warrant further research to expand understanding of the factors and barriers, which influence discharge in order to inform policy regarding transitions in care to the community. Despite an increase in the literature on this topic in recent years, there is still a need for better understanding of the complexity of the discharge process and the factors and barriers, which can influence discharge. This includes the need for more research on post-discharge outcomes when transitioning from a LTCF back to the community.

CIHR implemented Canada’s Strategy for Patient-Oriented Research (SPOR) to address Canada’s struggles with turning influential research into high-quality and cost effective care [72]. Moving forward there is a clear need for more patient centred research in regards to transitions from LTCFs to the community and emphasis placed on measuring whether the person in fact experienced improve health, wellbeing, and QOL. While there is an understanding of measurable post-discharge outcomes such as death, rehospitalization and readmission there is a need to better understand patients experiences and whether or not these discharge programs are successful from the point of the persons of focus and the perspectives of members of their circle of care including their informal caregiver(s). In addition, future research should investigate whether post-discharge outcomes would have/have not occurred, been prevented, or delayed had the person not transitioned out of the LTCF.

## Conclusion

This scoping review presents the scientific scenery of discharge out of LTCFs to community-based locations such as private homes, shared residences, or independent care. Most studies included older adult populations, were based in the USA, and focussed on understanding residents’ perspectives. Gaps remain in the understanding of post-discharge outcomes such as effects on the person’s health, wellbeing and quality of life. Little is known about whether or not the discharge programs are successful from the person’s perspective and from the views of members of their circle of care. To create best practices in this area, future research needs to expand understanding of the barriers and facilitators of resident transitions and provide more detailed description of subjective and objective post-discharge outcomes. Additionally, there is need for replication of study findings and to expand generalizability of findings beyond small geographic regions in the USA. With such evidence, the option to transition from LTCFs to the community can be further developed.

## Abbreviations

LTCF: Long term care facility
QOL: Quality of life
SNF: Skilled nursing facility
VA: Veteran Affairs
