# Correction to: factors affecting residents transition from long term care facilities to the community: a scoping review

**DOI:** 10.1186/s12913-017-2636-y

**Published:** 2017-10-30

**Authors:** Shannon Freeman, Kristen Bishop, Lina Spirgiene, Erica Koopmans, Fernanda C. Botelho, Trina Fyfe, Beibei Xiong, Stacey Patchett, Martha MacLeod

**Affiliations:** 1 School of Nursing, University of Northern British Columbia, 3333 University Way, Prince George, BC V2N 4Z9 Canada; 2 Faculty of Health Sciences, Health and Rehabilitation Sciences, Western University, 1151 Richmond St, London, ON N6A 3K7 Canada; 3 Department of Nursing and Care, Lithuanian University of Health Sciences, Mickevičiaus 9, -44307 Kaunas, LT Lithuania; 4 School of Health Sciences, University of Northern British Columbia, 3333 University Way, Prince George, BC V2N 4Z9 Canada; 5 School of Public Health, University of Sao Paulo, Dr. Arnaldo Street 715, Sao Paulo, Brasília, SP 01246-904 Brazil; 6 Northern Medical Program, University of Northern British Columbia, 3333 University Way, Prince George, BC V2N 4Z9 Canada; 7 School of Nursing, Jilin University, 965 XinJiang Street, ChangChun, JiLin, ChangChun, 130012 China; 8 Department of Quality, Planning and Information, Northern Health, 543 Front Street, Quesnel, BC V2J 5K7 Canada

After the publication of the article [1] it came to our attention that the author name Fernanda C. Botelho was incorrectly included as Fernanda C. Bothelo. The correct spelling of the name is in the authors list of this correction and updated in the original article.

## References

[1] Freeman S (2017). Factors affecting residents transition from long term care facilities to the community: a scoping review. BMC Health Serv Res.

